# Neural Mechanisms by Which Attention Modulates the Comparison of Remembered and Perceptual Representations

**DOI:** 10.1371/journal.pone.0086666

**Published:** 2014-01-21

**Authors:** Bo-Cheng Kuo, Duncan E. Astle

**Affiliations:** 1 Department of Psychology, National Taiwan University, Taipei, Taiwan; 2 Medical Research Council Cognition and Brain Sciences Unit, Cambridge, United Kingdom; University of California, San Francisco, United States of America

## Abstract

Attention is important for effectively comparing incoming perceptual information with the contents of visual short-term memory (VSTM), such that any differences can be detected. However, how attentional mechanisms operate upon these comparison processes remains largely unknown. Here we investigate the underlying neural mechanisms by which attention modulates the comparisons between VSTM and perceptual representations using functional magnetic resonance imaging (fMRI). Participants performed a cued change detection task. Spatial cues were presented to orient their attention either to the location of an item in VSTM prior to its comparison (retro-cues), or simultaneously (simultaneous-cues) with the probe array. A no-cue condition was also included. When attention cannot be effectively deployed in advance (i.e. following the simultaneous-cues), we observed a distributed and extensive activation pattern in the prefrontal and parietal cortices in support of successful change detection. This was not the case when participants can deploy their attention in advance (i.e. following the retro-cues). The region-of-interest analyses confirmed that neural responses for successful change detection versus correct rejection in the visual and parietal regions were significantly different for simultaneous-cues compared to retro-cues. Importantly, we found enhanced functional connectivity between prefrontal and parietal cortices when detecting changes on the simultaneous-cue trials. Moreover, we demonstrated a close relationship between this functional connectivity and *d′* scores. Together, our findings elucidate the attentional and neural mechanisms by which items held in VSTM are compared with incoming perceptual information.

## Introduction

The ability to detect changes in an ever-changing visual environment is important in daily life. Change detection is particularly challenging, because it requires the interface between perception, which is limitless in the amount and complexity of its contents, and visual short-term memory (VSTM), which is highly capacity limited [1]–[8]. Controlled attention is likely instrumental in comparing the perceptual and mnemonic representations. This is especially the case in demanding circumstances, such as when there are many items to search for a potential change. However, how attentional mechanisms operate upon the perceptual and VSTM representations and modulate their comparison remains largely unknown. Here we investigate the underlying neural mechanisms that control the allocation of spatial attention in a change detection paradigm, using functional magnetic resonance imaging (fMRI).

Neuroimaging evidence has shown that successful detection of a changed stimulus from the visual environment relies upon a distributed brain network including frontal, parietal, and temporal-occipital regions, relative to correct rejection or change blindness [9]–[15]. These studies particularly highlighted the importance of parietal cortex in change detection. For example, disrupting activity in the right posterior parietal cortex adversely affects the ability to detect changes of face stimuli [9]. This evidence is consistent with the view that the posterior parietal activity relates directly to the capacity limit of VSTM [16]–[18] and/or the attentional resources needed for VSTM rehearsal [19]. One strong possibility is that prefrontal and parietal regions act as the sources for generating top-down attentional signals, which bias neural activity in sensory-related brain regions [20]–[25], thereby supporting the attentional demands of detecting changes.

Previous studies have revealed that attention can modulate both perceptual and VSTM representations according to behavioural expectations or current task-goals 8,26–28. An influential neural theory of attention proposes that top-down attention biases the competition arising when different inputs compete for representation [21], [22], [29], [30]. Similar top-down attention biases can operate upon remembered information in VSTM [31]–[33]. Studies introducing retrospective cues (retro-cues) to shift attention to a specific item or location during VSTM maintenance can result in substantial improvements in retrieval accuracy and speed [8], [28], [34]–[39]. This type of spatial cue provides prior knowledge of a specific target item or location that needs to be compared to the upcoming probe or probe array. Recent fMRI investigations also demonstrated that attentional modulation of VSTM contents, using these kinds of cue, can influence the magnitudes or the patterns of neural activity in the posterior visual regions [40]–[49]. This modulation in visual activity may act to facilitate the comparison of the remembered item and the expected probe.

Although the remembered representations can be biased by shifts of attention, this competitive advantage is not observed whilst cueing simultaneously with the presentation of the probe stimuli (termed simultaneous-cue in this study or post-cue in others) [8], [38], [50]–[54]. This may be because the retro-cue enables participants to insulate or protect the VSTM item, such that its otherwise fragile representation is not overwritten by the onset of the subsequent competing probe array [8], [35], [38]; the simultaneous-cue is by definition too late to act in this way, so those attentional mechanisms must be recruited, once the interference has occurred. In short, the simultaneous cue requires participants to directly resolve the VSTM-perceptual interference at the cued location and detect any changes, whereas this is not necessary in the retro-cue condition.

In this study, we investigate the neural mechanisms by which attention modulates this comparison processes in a cued change detection task. Spatial cues were presented to shift attention either to the location of an item in VSTM prior to (retro-cues), or simultaneously with the comparison process (simultaneous-cues). A no-cue condition was also included. Firstly, we attempted to look at the mechanisms by which attention can be oriented, following a retro-cue, to and item in VSTM [35], [39]. In particular we focused on how this anticipatory attentional control will influence the neural responses by which VSTM and perceptual representations are compared (i.e. the effect of retro-cueing on change detection). Secondly, we examined the neural correlates of perceptual and VSTM comparison for the simultaneous-cues. Unlike retro-cues, simultaneous-cues do not allow for this anticipatory circumventing of the interference between perceptual and mnemonic representations. For this reason they are unlikely to yield as good a behavioural advantage as retro-cues [8], [38], and furthermore they will likely recruit attentional control mechanisms as participants attempt to resolve the competition necessary to detect changes. The aim of our design was to identify these neural mechanisms, and to understand their relationships with behaviour: we also test whether shifts of attention during the presentation of the probe stimuli involve changes in the functional interaction between prefrontal and parietal regions using a Psychophysiological Interaction (PPI) procedure [55], [56]. If this kind of mechanism is involved in the implementation of attentional control necessary to detect changes, then we expect the strength of functional coupling to influence behavioural performance.

## Materials and Methods

### Participants

All participants were right-handed according to the Edinburgh handedness inventory [57]. Fourteen healthy volunteers with normal or corrected-to-normal visual acuity were recruited from the undergraduate and graduate students at National Taiwan University and National Taiwan University of Science and Technology. Written informed consent was obtained from all participants prior to the study, and they were financially reimbursed for their time. We analysed data from 13 participants (age range 20–30 years, 6 females) because one participant showed excessive head movement in the scanner (greater than 5 mm). All experimental methods and procedures used in the study were non-invasive and had ethical approval from the Research Ethic Office of National Taiwan University.

### Task design

**Figure 1:**
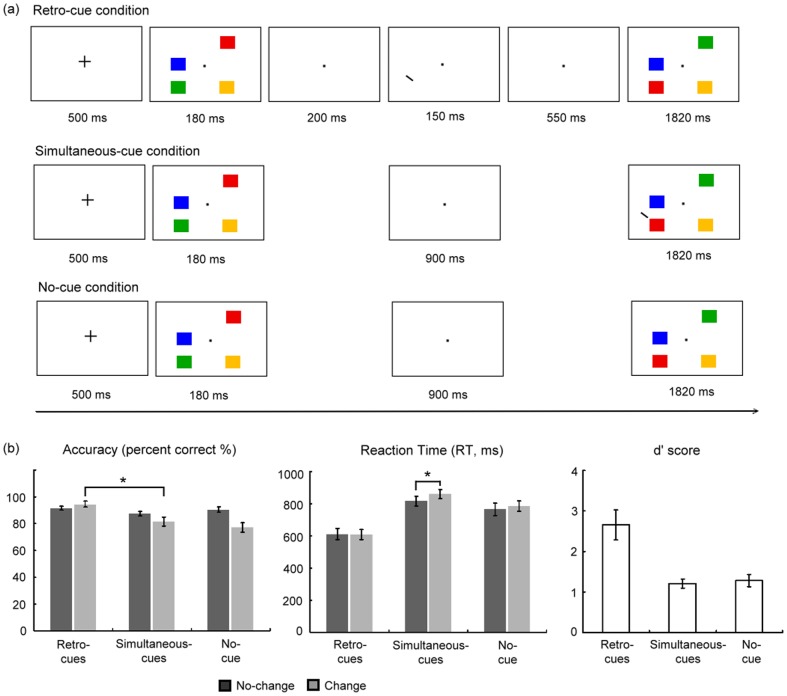
Schematics of experimental trials and behavioural results. (a) An example of a change trial in the retro-cue condition (top panel), simultaneous-cue condition (middle panel), and no-cue condition (bottom panel). The spatial cue consisted of a white line pointing to the upper left corner of a stimulus location and a grey background was used throughout the experiment (the cue was represented in black and background was represented in white in figure). (b) Behavioural results of successful change detection (change trials) and correct rejection (no-change trials) for accuracy (percent correct %) (left), reaction time (RT, ms) (middle), and *d′* score (right). Error bars represent standard errors of the means.

The experimental design followed a 3 (cue type: retro-cue, simultaneous-cue, and no-cue)×2 (change type: change and no-change) within-subjects factorial design. The task procedure is shown in Figure 1. Participants viewed a memory array, followed by a retention interval, and later by a probe array [3]. Their task was to identify any changes that occurred from one array to the next. A spatial cue was present during retention interval in the retro-cue condition or during the probe array in the simultaneous-cue condition. Participants simply had to detect changes without the aid of a spatial cue in the no-cue condition.

Each trial began with the onset of a fixation cross (500 ms duration), which signaled the onset of the trial. After that, participants viewed a memory array containing four different peripheral colour squares (180 ms duration). Participants were instructed to remember as many items as possible that were presented within the memory array. Following a retention interval (900 ms duration), the probe array was presented for 1,820 ms. In the retro-cue condition, a spatial cue was presented 200 ms after the offset of the memory array for 150 ms during the retention interval. In the simultaneous-cue condition, a spatial cue simultaneously appeared on the presentation of the probe array for 1820 ms. Participants' task was to decide whether the cued item of the probe array had changed relative to the preceding memory array in both retro- and simultaneous-cue conditions. In the no-cue condition, the participants' task was to judge whether a change had occurred between the probe array and the memory array. The same stimuli always appeared in the memory and probe arrays; however, two stimulus items always swapped locations on change trials (50%). The stimuli in the memory array and probe array were identical on no-change trials (50%). The inter-trial interval was presented for 3,000, 6,000, or 9,000 ms randomly so that the inter-trial interval was jittered to isolate the hemodynamic responses of individual trials [58].

### Stimuli

Stimulus arrays were composed of four coloured squares with the colours randomly selected from a set of six colours: red, green, yellow, blue, cyan, and magenta. Each stimulus item subtended a visual angle of approximately 0.52°×0.52° (edge-to-edge) and was positioned randomly in one of the eight possible peripheral locations of an invisible 3×3 matrix that subtended approximately 6.2°×6.2°. The spatial cue consisted of a white line pointing to the upper left corner of a stimulus location in the retro-cue and simultaneous-cue trials. When two arrays were identical on no-change trials, this cued position was randomly selected from the four stimulus locations. On change trials, the two changed items in the probe array were randomly selected from the four stimuli on each trial and the cued position was randomly selected from the two locations where changes would occur. The retro-cues and simultaneous-cues were always informative of the change location (i.e. they were 100% valid). Participants were instructed that a spatial cue indicated the location where a change would occur in change trials and were encouraged to use the cue information. A gray background was used throughout the experiment.

### Experimental procedure

The experimental session consisted of three runs (retro-cue, simultaneous-cue, and no-cue conditions), with 96 trials in each run, with the order randomised for each participant. In each run, half of the trials were the no-change trials and the other half were the change trials. Participants made change and no-change responses by pressing left button and right button on a button box using their right hand. Participants were instructed to respond as accurately and quickly as possible and maintain fixation on a small fixation marker at the centre of the screen during the presentation of the visual stimuli. Prior to fMRI scanning, participants completed one practice session (30 trials) outside of the scanner to ensure that they could perform the task as instructed. Ten practice trials contained retro-cues, 10 trials contained simultaneous-cues, 10 trials contained no cue, with half being change trials and half being no-change trials. Stimuli were presented using Presentation software (Neurobehavioral Systems, Inc., CA), and synchronized with the MRI scanner acquisition.

### fMRI acquisition and scanning parameters

This experiment was run on a Bruker MedSpec 3T system (Bruker, Ettlingen, Germany) at National Taiwan University with a quadrature birdcage head coil. The stimuli were visually presented on a goggle display system (Resonance Technology Inc., CA, USA). If required, both eyes were corrected to normal visual acuity with lenses during the experiment. Behavioral responses were recorded using an MRI-compatible fiber-optic light-sensitive response button box held in the participant's right hand. A single-shot T2*-weighted gradient echo-planar imaging (EPI) sequence (TR = 1500 ms, TE = 30 ms, flip angle = 90°) was used to measure the blood-oxygen-level-dependent (BOLD) signal. Functional images were obtained from 16 contiguous axial-oblique slices (thickness = 5 mm and gap = 1 mm), which were acquired parallel to the anterior commissure-posterior commissure (AC-PC) line and in plane resolution of 3.75×3.75 mm. The experiment was divided into 3 runs, each with 592 volumes. High resolution anatomical images were acquired by the RARE sequence (matrix size = 256×256 and FOV = 30×30 cm).

### Behavioural analyses

Behavioural measures, including accuracy (percent correct) and mean RTs, were firstly analysed by a 3 (cue type: retro-cue, simultaneous-cue, and no-cue)×2 (change type: change and no-change) repeated-measures analysis of variance (ANOVA) to assess change detection performance. We also analysed the behavioural data in a 2 (cue type: retro-cue, simultaneous-cue)×2 (change type: change and no-change) repeated-measures ANOVA and tested for the difference in attentional modulation in change detection. Only correct responses were included for RT analyses. We also analysed a sensitivity score for the successful detection/correct rejection discrimination [*d′* = *Z*(hit rate)−*Z*(false alarm rate)] [59], in a one-way (cue type: retro-cue, simultaneous-cue, and no-cue) repeated-measures ANOVA. Hit rate was defined as the conditional probability that the participants responded “change” when the target item was different, and the false-alarm rate was defined as the conditional probability that the participants responded “change” when the target item was the same.

### fMRI data analyses

Three types of analysis were performed on the fMRI data. A whole-brain univariate analysis was used to identify brain areas activated based on the interaction between cue-type (retro-cues, simultaneous-cues, and no-cue) and change detection (change versus no-change). Region of interest (ROI) analyses were then conducted to test the modulation in activity in both retro-cue and simultaneous-cue condition. To avoid circular analysis (or double dipping) [60], we used the no-cue condition as an independent scan to localise functional ROIs in parietal and visual cortices. Finally, we used PPI to examine the relationship between the functional correlation between the prefrontal and posterior parietal areas and successful change detection (focussing particularly on simultaneous cues).

The fMRI data processing and analyses were carried out with SPM5 software (Wellcome Trust Centre for Neuroimaging, University College London, UK) in MATLAB (The MathWorks). The first eight volumes of each run were discarded to allow for magnetic saturation effects. The remaining functional images were corrected for head movement artefact and timing differences in slice acquisitions. Pre-processed functional images were then co-registered to the individual anatomical image and normalised to the standard SPM/MNI brain template [61] and resampled to a 2-mm isotropic voxel size. Normalised images were spatially smoothed with a Gaussian kernel of 8-mm full width at half maximum (FWHM) to accommodate any anatomical variability across participants [62], [63]. The time-series data were then high-pass filtered with a frequency cut-off at 128 s and prewhitened by means of an autoregressive model AR(1).

In a whole-brain univariate analysis, statistical inference was based on a random-effect approach at two levels. At the individual level, the data of each participant were analysed using the general linear model (GLM) by fitting the time series data with the canonical hemodynamic response function (HRF) modeled at the events of interest. Only the events with correct responses were modeled for each of the 6 conditions: 3 (cue type: retro-cue, simultaneous-cue, and no-cue)×2 (change type: change and no-change). Linear contrasts were computed to characterise responses of interest. We firstly computed the contrasts of successful detection (change trials) versus correct rejection (no-change trials), for retro-cue, simultaneous-cue and no-cue trials, respectively. Moreover, we computed three interaction contrasts: (1) ‘simultaneous-cues: change versus no-change’ versus ‘retro-cues: change versus no-change’; (2) ‘simultaneous-cues: change versus no-change’ versus ‘no-cue: change versus no-change’; and (3) ‘retro-cues: change versus no-change’ versus ‘no-cue: change versus no-change’. The estimations for each contrast were then entered into a standard SPM group-level analysis in which the participants were treated as a random variable using a one-sample *t* test. We used a threshold of *p*<0.05, correcting for multiple comparisons using family-wise error rate at the cluster level.

In ROI analyses, we used the main effect of the no-cue condition from the group-level analysis, collapsing across change and no-change trials to define two functional ROIs in the occipital cortex [Talairach coordinates (*x*, *y*, *z*): left occipital ROI = −34, −83, 20; right occipital ROI = 31, −79, 16]. We also used the contrast of change versus no-change trials in the no-cue condition from group-level analysis to determine four functional ROIs in the parietal cortex [Talairach coordinates (*x*, *y*, *z*): left superior parietal lobule (SPL) = −18, −58, 56; right SPL = 23, −62, 54; left intraparietal sulcus (IPS) = −48, −33, 49; right IPS = 33, −47, 42]. The size of each ROI was defined as a sphere with its centre of the predetermined coordinates with a radius of 6 mm. Mean beta estimates for the effects of interest were extracted for all conditions for each participant using MarsBaR (http://marsbar.sourceforge.net/). We then tested for modulatory effects in a 2 (cue type: retro-cue, simultaneous-cue)×2 (change type: change, no-change) repeated-measures ANOVA for each occipital and parietal ROI.

Finally, we identified cortical control areas that may interact with parietal cortex using a PPI analysis. Specifically, we tested change-detection-related activity in brain regions that were functionally correlated with those in the parietal cortex for the change trials in contrast to no-change trials in the simultaneous-cue condition. We did not test functional connectivity for the retro-cues given no significant effect of change detection was observed in a whole-brain univariate analysis (see fMRI results for details). According to previous evidence [10], [12], we selected a seed region (left IPS) in the parietal cortex based on the conjunction of both the interaction results from the contrast of ‘simultaneous-cues: change versus no-change’ versus ‘no-cue: change versus no-change’ and the contrast of ‘simultaneous-cues: change versus no-change’ versus ‘retro-cues: change versus no-change’. For each participant, we defined the size of the seed regions as a sphere with its centre on the predetermined coordinates with a radius of 6 mm. The deconvolved time-series data for the seed voxels was extracted from each participant. The product of these time-series data and the psychological vector of interest (‘change versus no-change’) in the simultaneous-cue condition, resulted in the psychophysiological interaction term, which was convolved with the HRF [56]. The physiological variable (Y regressor), the psychological variable (P regressor) and their interaction (PPI regressor) were computed for each participant as regressors. The estimates for PPI at the individual level were then entered into random-effect group-level analysis with a threshold of *p*<.05, controlling for family wise error rate with a nonparametric permutation analysis (http://www.fil.ion.ucl.ac.uk/spm/snpm) [64].

All significantly activated areas were then transformed into the Talairach space [65], using a modified version of the mni2tal MATLAB script (http://imaging.mrc-cbu.cam.ac.uk/imaging/MniTalairach). Anatomical localisations were done with Talairach Daemon software (Research Imaging Center, Health Science Center, San Antonio, TX).

## Results

### Behavioural results

Participants performed reliably well, but did not reach ceiling performance (Figure 1b). The 3 (cue type: retro-cue, simultaneous-cue, no-cue)×2 (change type: change, no-change) repeated-measures ANOVA revealed a significant main effect of cue type on accuracy [*F*(2, 12) = 16.79, *p*<.005], showing more accurate performance for retro-cues (93.03%±6.61) compared to simultaneous-cues (84.46%±9.57) and no-cue trials (83.73%±11.94). Change type also significantly interacted with cue type [*F*(2, 24) = 7.03, *p*<.005]. This interaction arose because the effect of cue type was not significant on no-change trials (*ps*>.1) whereas accuracy for retro-cues trials (94.55%±7.63) was significantly higher than simultaneous-cues (81.57%±11.96) and no-cue trials (77.08%±12.90) (*p*<.005) on change trials. Converting the accuracy data into *d′* scores revealed a significant main effect of cue type [*F*(2, 12) = 14.04, *p*<.005], with higher *d′* scores for retro-cue (2.66±1.29 *d′* scores) compared to simultaneous-cue (1.21±0.38 *d′* scores) and no-cue trials (1.28±0.56 *d′* scores). Finally, the analysis on RT data indicated a faster RT for retro-cue (609.26 ms±117.60) compared to simultaneous-cue (838.83 ms±100.02) and no-cue trials (775.17 ms±121.16) [*F*(2, 12) = 35.45, *p*<.005].

To test for the difference in attentional modulation of change detection performance, we computed a 2 (cue type: retro-cue, simultaneous-cue)×2 (change type: change and no-change) repeated-measures ANOVA and showed a significant main effect of cue type on accuracy [*F*(1, 12) = 22.96, *p*<.005], indicating more accurate performance for retro-cues compared to simultaneous-cues. The interaction of change type and cue type was marginally significant [*F*(1, 12) = 4.33, *p* = .059]. Follow-up comparisons indicated more accurate performance for retro-cues compared to simultaneous-cues when changes occurred [*t*(12) = 3.84, *p*<. 005] but only marginally more accurate responses for retro-cues (91.51%±5.61) compared to simultaneous-cues (87.34%±6.10) when no change occurred [*t*(12) = 1.96, *p* = .07]. Furthermore, RT data revealed faster RTs for retro-cue compared to simultaneous-cue trials [*F*(1, 12) = 65.78, *p*<.005]. A significant interaction of change type and cue type was observed [*F*(1, 12) = 5.30, *p*<.05]. This interaction demonstrated a slower RT for change trials (860.97 ms±106.15) compared to no-change trials (816.68 ms±106.83) in the simultaneous-cues condition (*p*<.05) but not in the retro-cues condition (*p*>.1).

In summary, the behavioural data revealed that participants could use the retro-cues to orient their attention to items maintained in VSTM, thereby enabling them to detect changes more reliably and more quickly. This benefit is specific to the anticipatory orienting that the retro-cue affords; the same benefit is not seen for simultaneously presented cues.

### fMRI results

#### Whole-brain univariate results

**Figure 2:**
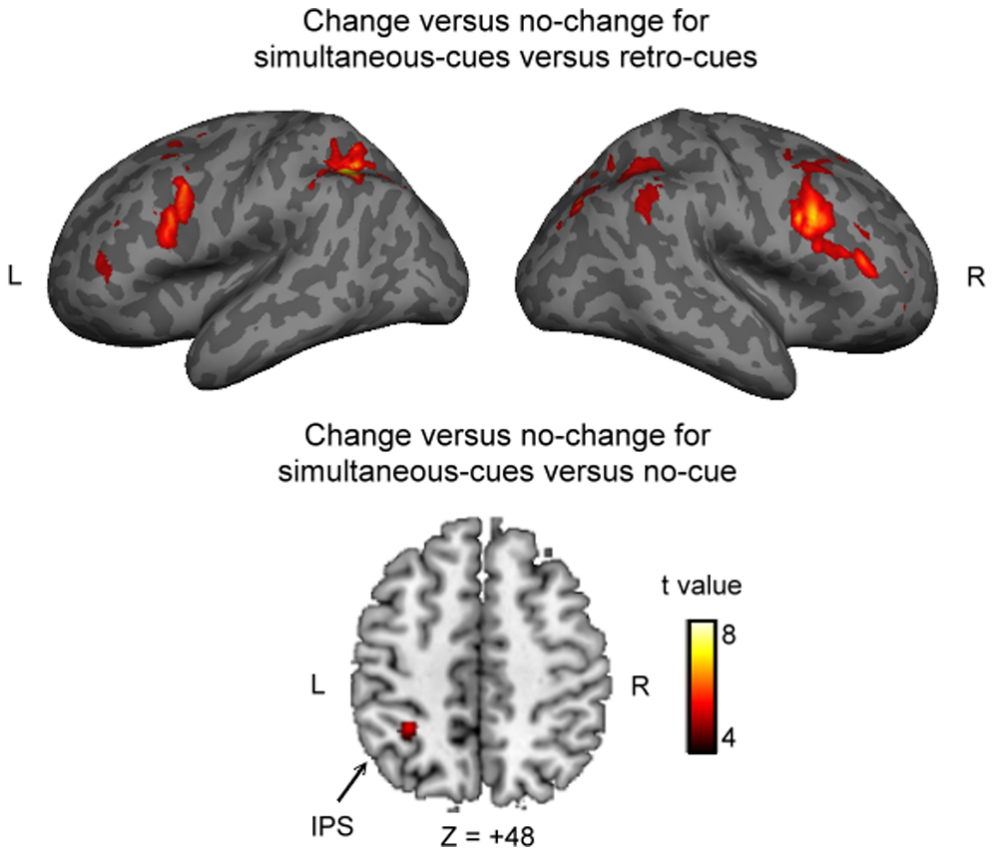
Whole-brain univariate results. Areas of significant activation were associated with successful change detection (change trials) in contrast to correct rejection (no-change trials) for the contrast of the simultaneous-cues versus retro-cues (top panel) and for the contrast of the simultaneous-cues versus no-cue (bottom panel, IPS: intraparietal sulcus).

We firstly tested for the interaction results from the contrast of ‘simultaneous-cues: change versus no-change’ versus ‘retro-cues: change versus no-change’ (Figure 2 and Table 1). We found a distributed and extensive activation pattern in cortical regions including middle and inferior frontal gyrus (MFG and IFG), frontal eye fields (FEF), ventrolateral prefrontal cortex (VLPFC), and anterior cingulate cortex (ACC) within the frontal cortex. We also observed significant bilateral activation in the SPL, inferior parietal lobule (IPL) and IPS, and precuneus within the posterior parietal cortex. Moreover, subcortical regions including thalamus and putamen were activated. These results were in line with previous findings that identify activity in a prefrontal and parietal neural network [9]–[14]. Finally, we tested for the interaction of cue type (simultaneous-cues and no cue) and change type and identified a significant activation in the left IPS (peak coordinates = −30, −55, 41) (Figure 2). No significant activation was observed when the same comparison was done for retro-cue versus no-cue trials.

**Table 1:** Brain areas and their Talairach coordinates (*x*, *y*, *z*) significantly activated by the interaction of successful detection on change trials with correct rejection on no-change trials for simultaneous-cues versus retro-cues.

Brain areas		*x*	*y*	*z*	*t*
Anterior cingulate cortex	Med	9	29	39	6.78
Frontal eye fields	L	−22	14	47	5.23
	R	35	10	58	7.75
Middle frontal gyrus	L	−48	30	24	4.95
	R	49	21	33	4.49
Inferior frontal gyrus	L	−40	11	27	7.28
	R	47	10	23	8.44
Ventrolateral prefrontal cortex	L	−34	39	3	4.94
	R	41	50	1	5.21
Superior parietal lobule	L	−30	−47	41	9.67
	R	29	−64	51	6.12
Inferior parietal lobule/intraparietal sulcus	L	−46	−37	38	5.80
	R	33	−51	44	5.83
Precuneus	L	−26	−61	38	5.61
	R	19	−59	41	4.77
Thalamus	L	−16	−6	2	6.67
	R	13	−4	5	5.41
Putamen	L	−20	2	10	4.58
	R	23	4	8	5.69

Note: Medial: Med; Left hemisphere: L; Right hemisphere: R; *t*: t-score.

#### ROI analysis

**Figure 3:**
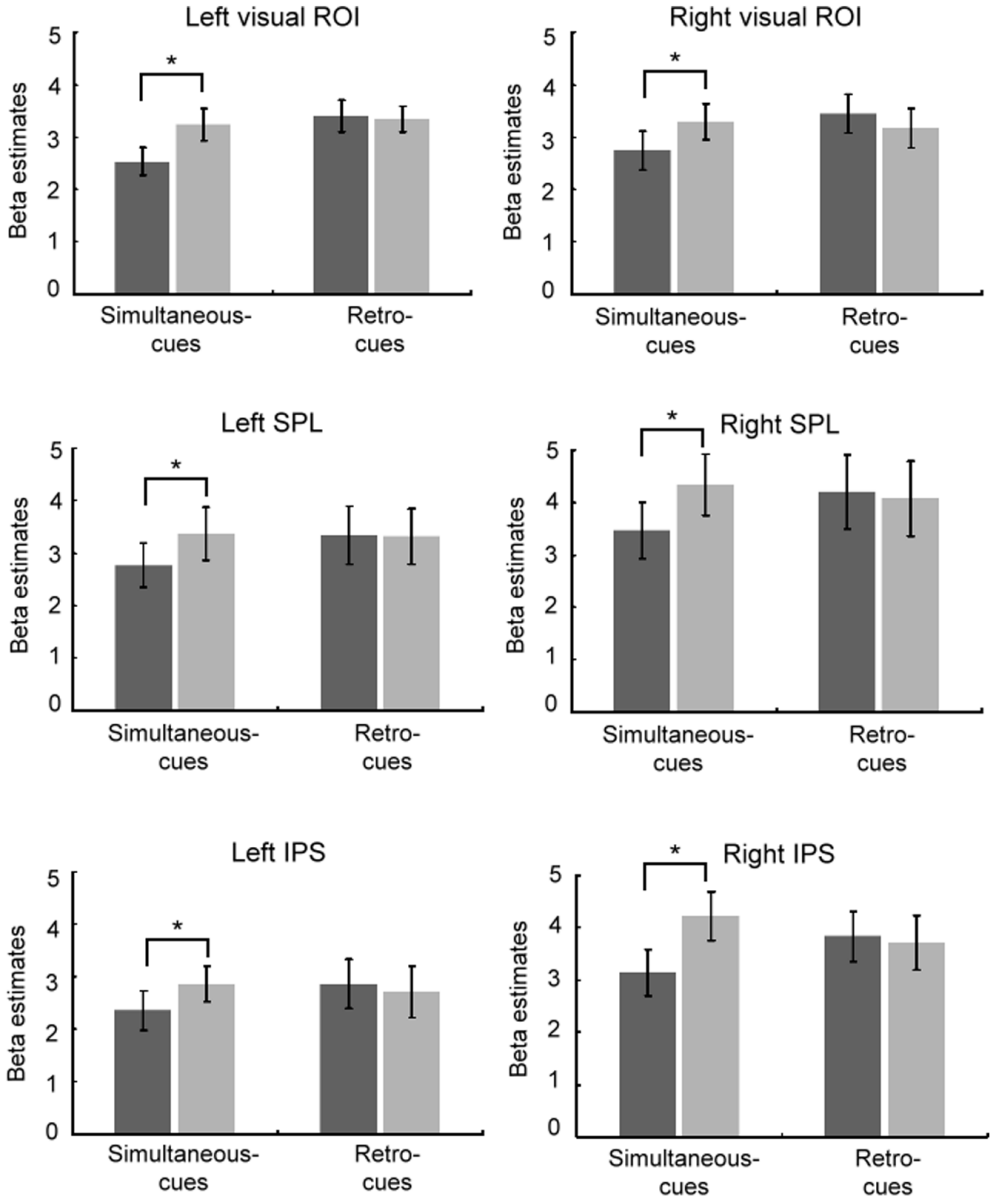
Region of interest (ROI) results in the posterior visual and parietal regions. ROIs were selected based on the no-cue condition. Successful change detection (change trials) yielded higher mean beta values than correct rejection (no-change trials) for simultaneous-cues, relative to retro-cues (SPL: superior parietal lobule; IPS: intraparietal sulcus). Error bars represent standard errors of the means.

The ROI results are illustrated in Figure 3. We tested for the presence of attentional modulation of activity in the visual and parietal ROIs for retro-cues and simultaneous-cues. An analysis of the ROI data in a two-way repeated-measures ANOVA revealed a significant interaction between cue type and change type in the left visual [*F*(1, 12) = 6.41, *p*<.05], right visual [*F*(1, 12) = 8.13, *p*<.05], left SPL [*F*(1, 12) = 5.92, *p*<.05], right SPL [*F*(1, 12) = 16.15, *p*<.005], left IPS [*F*(1, 12) = 5.72, *p*<.05], and right IPS [*F*(1, 12) = 28.12, *p*<.005]. Post hoc analyses indicated that successful detection yielded higher mean beta values compared to correct rejection in all visual and parietal ROIs for simultaneous-cues (*ps*<.05). However, there was no significant difference between successful detection and correct rejection for all ROIs for retro-cues (*ps*>.1). These ROI results not only confirmed the whole-brain univariate results but also indicated the modulatory effect in parietal and visual occipital regions.

#### Functional connectivity analysis

**Figure 4:**
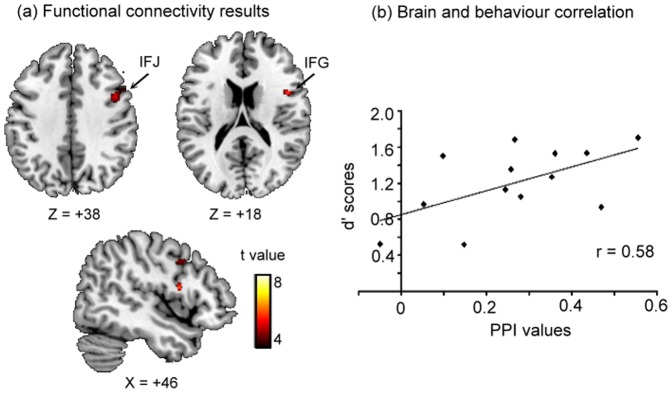
Functional connectivity results and brain-behaviour correlation. (a) Functional connectivity of attentional modulation of the comparison of remembered and perceptual representations for simultaneous-cues. These results showed functional couplings with right inferior frontal junction (IFJ) and inferior frontal gyrus (IFG) when left intraparietal sulcus (IPS) was a seed region. (b) A significant positive correlation was observed between the PPI estimates of functional connectivity in the right IFJ with left IPS and *d′* scores (Pearson's *r* = 0.58, *p*<.05).

Functional connectivity results are illustrated in Figure 4. Our main question was whether successful change detection relied on functional coupling between prefrontal and parietal regions in controlling of comparison process. We tested this hypothesis in a PPI analysis using the left IPS as a seed region and yielded significant effects with the right inferior frontal junction (IFJ) (peak coordinates = 43, 9, 36) and IFG (peak coordinates = 45, 8, 14) within the prefrontal cortex for simultaneous-cues.

#### A relationship between brain and behaviour

Finally, we tested for a relationship between the strength of functional connectivity and behavioural performance. The PPI estimates were extracted from the IFJ and IFG from each participant in the simultaneous-cue condition based on the contrast of change vs. no-change trials using Marsbar. Each frontal ROI was defined with the size as a sphere with its centre of the peak coordinates with a radius of 6 mm. We then examined Pearson's correlation between the PPI estimates and behavioural measures (*d′* scores and RTs). A significant positive correlation was observed between the PPI estimates of functional connectivity in the right IFJ with left IPS and *d′* scores (*r* = 0.58, *p*<.05) (Figure 4b). This brain and behaviour correlation suggests that participants with stronger functional coupling between the IFJ and IPS exhibit better behavioural performance in discrimination sensitivity for VSTM and perceptual comparisons. No other significant correlation was found in the IFG or with the RTs (*ps*>.1).

## Discussion

Using a cued change detection task, we presented cues during the initial maintenance or during the subsequent comparison. This enabled us to investigate the neural mechanisms by which attention is recruited in order to facilitate the VSTM and perceptual comparison process. Cues delivered in advance of the changes – retro-cues – enabled participants to deploy their attention in advance, conferring a large behavioural advantage over no-cue and simultaneous-cue trials. The whole-brain analysis showed a distributed and extensive activation pattern in the prefrontal and parietal cortices that mirrored the increased behavioural difficulty of successful change detection for the simultaneous-cues compared to the retro-cues. The ROI analyses using the no-cue condition as an independent functional localiser confirmed the difference in neural responses for change versus no-change trials in the visual and parietal ROIs for simultaneous-cues, relative to retro-cues. Moreover, we found a significant functional coupling between prefrontal and parietal cortices; activity in the right IFJ and IFG became strongly coupled to the left IPS when detecting changes on the simultaneous-cue trials. Finally, a close relationship between the PPI estimates of functional connectivity in the right IFJ with left IPS and *d′* scores was found, indicating the better behavioural performance with stronger connectivity. In sum, we were able to identify a set of neural mechanisms that mirrored our cueing manipulation. We suggest that these neural mechanisms reflect the allocation of attention to facilitate change detection. One possibility is that this occurs via the biasing of the competitive processing of the two types of representations (VSTM and perceptual), such that changes can be successfully detected, and that such a process is not necessary when attention has been deployed in advance.

### Retro-cues, but not simultaneous-cues, confer a change detection benefit

Consistent with behavioural evidence [28], [35], [37], [38], we observed a beneficial effect of retro-cues for both change and no-change trials, relative to simultaneous-cues or no-cue baseline. These benefits are in terms of RTs, accuracy and *d′* scores, suggesting the results were not due to a speed-accuracy trade-off or response bias. In contrast, we found no relative advantage for the simultaneous-cues. We suggest that the benefit of a retro-cue in this context is that it enables participants to overcome the interfering effect of subsequent perceptual stimuli on their fragile VSTM representation [8], [38], [50]–[54]. This probe interference is particularly evident when a changed item was presented.

In accordance with previous fMRI findings [9]–[14], our whole-brain univariate results found increased activation in a distributed cortical network. This included prefrontal and parietal regions, and subcortical regions including putamen and thalamus. This recruitment was specifically for successful change detection in contrast to correct rejection, and more so for the simultaneous-cue relative to the same contrast for the retro-cue condition. This pattern mirrors the increased attentional demands of comparing the VSTM and perceptual item at the simultaneously cued location, relative to doing this in the retro-cue condition. Furthermore, our ROI analyses confirmed this modulatory effect for change detection in the posterior visual and parietal regions [9], [10], [12], [18]. The use of ROIs, based on the no-cue condition as an independent functional localiser, ensures that the effects in the visual and parietal cortices that we observed result from the cueing modulation of the comparison process. Taken together, our whole-brain and ROI results suggest that activity of the posterior parietal and visual areas was influenced by the requirements of the type of cue. This was only the case for spatial cues presented *simultaneously* with the presentation of the probe array, implying that this mechanism of top-down modulation is not specific to the orienting of attention per se (as this also occured in the retro-cue condition), but indexes the use of attention to deal with the interfernce imposed by the subsequent perceptual information. Given its neural correlates, it is perhaps surprising that the simultaneous-cues do not confer a change detection benefit over the neutral baseline. We suggest that the way in which participants detect changes when they have no specific location to search is rather different. When the simultaneous-cue appears, we suggest that participants engage in a focussed attempt to resolve the interference between the new probe item and the old VSTM item, such that any changes can be detected; this is what we think our neural responses reflect. However, in the block with no-cues participants may adopt some alternative approach, whereby they search all locations in parrallel – essentially searching for a pop-out [2]. In behavioural terms it may not be that one is any better than the other. Nonetheless, we wanted to demontrate that there was some behavioural consequence of the simultaneous cueing, by relating our functional connectivity measures to detection sensitivity, which we discuss in the subsequent section.

The modulation of visual activity during attentional control tasks is in line with previous findings in both perceptual and VSTM domains. It has been shown that attention modulates visual processing by enhancing neural responses to attended stimuli or suppressing responses to unwanted information when multiple visual stimuli items appear simultaneously in the visual field [66]–[69]. Also, directing attention to a target location from the visual environment in anticipation of a behavioural relevant stimulus can increase subsequent neural responses in visual areas [70], [71]. In parallel to the modulation in perceptual domain, attention can also modulate visual activity in supporting and protecting VSTM representations from inter-item competition or from the probe interference [33], [39], [45].

In summary, drawing together our behavioural and functional imaging results, we demonstrate that the modulation of visual activity during change detection mirrors the increased difficulty of comparing perceptual and VSTM representations in the simultaneous-cue condition. This is especially evident when subjects need to correctly detect a change in the environment (relative to no-change trials). Furthermore this modulation is only apparent in the simultaneous-cue condition – when attention is oriented in advance, this difficulty is overcome, and the same neural mechanisms are not recruited.

### The role of the SPL and IPS in attentional control and VSTM

The SPL and IPS have been characterised as carriers of the top-down attention signals in both perceptual and memory domains [20], [21], [25], [72]. Neuroimaging studies have demonstrated that a transient increase in SPL activity accompanies shifts of attention between two peripheral spatial locations [73], [74], two voices [75], and visual and auditory domains [76]. Recent evidence from patients with SPL lesion indicates a causal role in goal-directed shifts of attention [77]. However, studies have shown that these areas are implicated not only in attentional control processes per se, but also in VSTM. For example, SPL lesions also result in impairments in the manipulation and rearrangement of internal information in VSTM [78]. Similarly, activity in the IPS is consistently observed when attention is explicitly oriented to cued items within VSTM [49], [79], [80]. IPS activity reflected the amount of stored information and the magnitude of activity was correlated with an individual's VSTM capacity [16]–[18]. So there is some controversy as to whether the involvement of these parietal areas in VSTM tasks is because of mnemonic processes per se, or because of the inherent attentional demands of the task. A recent fMRI investigation has argued that activity in the IPS represents the attentional demand of rehearsal processes that changes as a function of the length of VSTM retention interval (e.g. long versus short delay) [19]. They showed greater IPS activity as the attentional demands increased (long delay with the increase in VSTM load). Consistent with this account, we showed the modulation of these areas with simultaneous cueing. When attention was shifted *prior* to the comparison process, participants were presumably able to bias the competitive processing, and insulate the cued VSTM items from probe interference. Accordingly, retro-cues did not produce the same change-related activity as was present with simultaneously presented cues. The difference in activation between change versus no-change in the SPL and IPS therefore appears to reflect the regulation of the comparison of the inconsistent VSTM and perceptual representations for successful change detection.

Of particular interest in this study was that the functional connectivity analysis revealed inter-regional correlation between right prefrontal regions and the left IPS in supporting of the comparison process for the simultaneous-cues. Furthermore, the strength of this connectivity was associated with behavioural performance. As noted above, it is generally accepted that the prefrontal and parietal cortices provide top-down biasing signals in favour of those inputs most relevant to our expectations and goals [23], [24], [81]. These attentional biasing signals can also guide selection or orienting in VSTM [31], [49]. A series of investigations by Gazzaley and colleagues showed that the strength of functional coupling between the right IFJ and category-specific visual areas (e.g. colour or motion) was influenced by the magnitude of the attentional enhancement for task-relevant items and suppression of irrelevant stimuli during VSTM encoding [82]–[84]. Our findings add significantly to these previous demonstrations of inter-regional correlation between regions. We demonstrate a close relationship between functional connectivity in neural responses and discrimination sensitivity in behavioural performance, specifically in the context of detecting changes in the environment.

An alternative interpretation is that our effects may be driven by eye-movements. Whilst some findings indicate less of a neural overlap between attention and eye movement control than previously thought [85], [86], there remain a number of studies demonstrating a functional and anatomical overlap in dorsal frontal and parietal areas (e.g. FEF, SPL and IPS) between these two functions [87]. If our results were caused by eye movements, one might expect to find stronger retro-cueing effects in the eye movement-related areas, relative to simultaneous cues, for saccade preparation [88], [89]. However, we did not find any significant difference in activation between two cueing conditions. Moreover, it is important to remember that our principal result is significant activation in these regions for simultaneous-cues in contrast to retro-cues for successful change detection versus correct rejection. We do not think that this pattern of results can be explained by eye-movements. Also, our PPI analysis demonstrated a functional correlation between IFJ and IPS instead of eye movement-related areas (e.g. FEF or SPL) for the simultaneous-cues.

In conclusion, we suggest that top-down attentional mechanisms can optimise task-relevant information during multiple domains of processing - perception, VSTM, and their comparisons. We particularly highlight the roles of attentional control mechanisms in biasing the competitive process of comparing remembered and perceptual representations. When subjects direct their spatial attention to a location and attempt to compare a previously seen item to the new item just presented there, we observed enhanced change-related activity and functional coupling between the IPS and IFJ. Furthermore, the strength of this coupling reflected participants' sensitivity to detect such changes.
